# A novel sequential risk assessment model for analyzing commercial aviation accidents: Soft computing perspective

**DOI:** 10.1111/risa.14486

**Published:** 2024-07-08

**Authors:** Amirhossein Nosrati Malekjahan, Ali Husseinzadeh Kashan, Seyed Mojtaba Sajadi

**Affiliations:** ^1^ Faculty of Industrial and Systems Engineering Tarbiat Modares University Tehran Iran; ^2^ Operations and Information Management Department Aston Business School Aston University Birmingham Birminghamm UK

**Keywords:** aviation accidents, FMEA, fuzzy cognitive map, intuitionistic fuzzy environment, risk assessment

## Abstract

Due to the importance of the commercial aviation system and, also, the existence of countless accidents and unfortunate occurrences in this industry, there has been a need for a structured approach to deal with them in recent years. Therefore, this study presents a comprehensive and sequential model for analyzing commercial aviation accidents based on historical data and reports. The model first uses the failure mode and effects analysis (FMEA) technique to determine and score existing risks; then, the risks are prioritized using two multi‐attribute decision making (MADM) methods and two novel and innovative techniques, including ranking based on intuitionistic fuzzy risk priority number and ranking based on the vague sets. These techniques are based in an intuitionistic fuzzy environment to handle uncertainties and the FMEA features. A fuzzy cognitive map is utilized to evaluate existing interactions among the risk factors, and additionally, various scenarios are implemented to analyze the role of each risk, group of risks, and behavior of the system in different conditions. Finally, the model is performed for a real case study to clarify its applicability and the two novel risk prioritization techniques. Although this model can be used for other similar complex transportation systems with adequate data, it is mainly employed to illustrate the most critical risks and for analyzing existing relationships among the concepts of the system.

## 1 INTRODUCTION AND LITERATURE REVIEW

The aviation industry is a cornerstone of modern society, providing efficient transportation services globally (Rieber, 1995; Srinivasan et al., 2019). Despite technological advancements and stringent safety measures, aviation accidents and incidents continue to pose a threat, leading to human casualties and substantial financial losses each year (Chandra, 2023). Analyzing these occurrences is a complex endeavor, as they involve a multitude of interconnected factors and conditions, compelling researchers to account for these intricate relationships (Shen, 2023; Zhang & Mahadevan, 2021). Airlines and aviation professionals invest significant resources in maintaining safety as a top priority, closely monitoring their performance, and implementing stringent regulations and safety systems (Huesler & Strobl, 2023; Valdés & Comendador, 2011). Ensuring aviation safety hinges on effectively addressing accidents and incidents by identifying their causal factors and taking preventive actions before major events occur (Dong et al., 2021).

In the aviation sector, preventive measures are essential to reduce accidents and incidents (Nazeri et al., 2008). To do this, a reactive approach, involving the analysis of past incidents, helps identify patterns of causal and effect factors linked to aircraft accidents. This proactive analysis enables the implementation of necessary actions to prevent and mitigate future occurrences (Fuller & Hook, 2020). Key factors encompass failures, errors, causes, and conditions that contribute to these events. Emphasizing this proactive approach can significantly decrease the frequency of accidents and incidents, enhancing aviation safety overall (Sayyadi Tooranloo et al., 2018).

In the subject of safety and reliability, risk assessment and failure analysis techniques are frequently used in various industries and scopes like new product development processes in the food industry (Sharifi et al., 2022), environmental risks in onshore and offshore drilling operations in the oil industry (Afzali Behbahani et al., 2022), and failure modes of wind turbines in a wind farm (Ghoushchi et al., 2022). In addition, these popular techniques are utilized to analyze aviation accidents, incidents, failure modes, and risks. The first part of research usually uses these mentioned methods, besides decision making, analytical tools like the analytic hierarchy process, and so on, to overcome the weaknesses of the measurement indicators like risk priority number (RPN), criticality degree, and enhance the outcomes. They aim at a particular scope, for example, a component of an aircraft like a landing gear system (Yazdi et al., 2017) and flaps system due to their vital role in the safety of the flight (Xiang et al., 2011) or even common failures in a particular industry like aerospace (Dandachi & El Osman, 2017). However, most of them usually look for the most critical failures based on a specific ranking obtained in the study procedure.

The next part of the research focuses on other sectors of the aviation industry, like the role of humans in civil aviation occurrences and the relationship between human error and aviation accidents (Li et al., 2008), which showed that the incorrect acts of staff are severely effective in this scope; or the role of airframe icing through a statistical review due to flying at high altitudes by aircraft and low air temperature conditions (Petty & Floyd, 2004). Aviators and researchers believe that a comprehensive safety management system (SMS) is a prerequisite for a reliable aviation network. This system contains precise and extensive concepts and instructions that should be implemented appropriately (Netjasov & Janic, 2008). There are accurate SMS systems in advanced countries like the United States, Canada, and the United Kingdom, which vulnerable countries can consider (Yeun et al., 2014). The crucial aspect of analyzing aviation accidents and incidents is evaluating the relationships between influential factors; fuzzy cognitive maps (FCMs) are one of the most popular tools for this purpose, but their combination with data mining to determine causes of aviation incidents (Aguilar et al., 2017) and analyzing airport risks based on experts’ opinions (Rezaee & Yousefi, 2018) and overall in this subject has been scant.

Risk assessment models and techniques have frequently been used to enhance safety in this industry (Patriarca et al., 2019), so that Bartulović and Steiner (2022) introduced some of these existing models. For example, Lee (2006) developed a quantitative model for evaluating aviation safety risk factors by integrating the fuzzy linguistic scale method, failure mode, effects, and criticality analysis principle; Stroeve et al. (2009) illustrated that Monte Carlo simulation can be employed as a useful model for accident risk assessment through safety‐relevant air traffic scenarios. In addition, Hadjimichael (2009) introduced the flight operation risk assessment system (see Table 1 for abbreviations), for proactive risk assessment in aviation, capable of assessing various flight operation mishap risks, including runway excursions and turbulence. Cohen et al. (1999) crafted a risk assessment model utilizing data from flight recorders (flight data recorder and quick access recorder) to pinpoint precursor events within aircraft performance conditions, thereby elevating the assessment of incident and accident risks. Analysis of the accidents and incidents in any crucial transportation system like civil aviation needs to be done considering all special requirements and features. This matter requires a model that considers all these features and has the best performance in this scope; this study introduces such a model that has considerable advantages rather to previous models and techniques. It should be mentioned that the model presented in this article needs access to accident reports, expert panels with sufficient experience and skills, and numerical calculations compared to other existing models. In Table 2, these features are specified, and it also shows which of the requirements are met by each of these popular models (Wienen et al., 2017) in previous studies.

**TABLE 1:** List of abbreviations.

Abbreviation	Explanation
AAIB	Aviation Accident Investigation Board
AHP	Analytic hierarchy process
ASN	Aviation Safety Network
ATC	air traffic control
CAA	civil aviation authority
CRM	cockpit resource management
CVR	Content validity ratio
FCM	fuzzy cognitive map
FDR	flight data recorder
FMEA	Failure mode and effects analysis
FORAS	Flight operation risk assessment system
FS	Fuzzy set
ICAO	International Civil Aviation Organization
IF‐FMEA	Intuitionistic fuzzy failure mode and effects analysis
IF‐RPN	Intuitionistic fuzzy risk priority number
IFSs	Intuitionistic fuzzy sets
ILS	Instrument landing system
MADM	Multi‐attribute decision making
RB‐VS	Ranking based on vague set
RPN	Risk priority number
SMS	Safety management system
QAR	quick access recorder

**TABLE 2:** Previous accident analysis models and techniques.

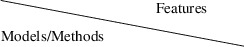						
	Comprehensive set of factors	Considering interactions among factors	Treatment of environmental uncertainties	Scoring and prioritizing risk (or factors)	Scenario analysis and prediction	Comprehensive and structured
FCM using data mining (Aguilar et al., 2017)	✓	✓	⨯	⨯	⨯	⨯
FCM using experts’ judgment (Rezaee & Yousefi, 2018)	⨯	✓	⨯	✓	⨯	⨯
Risk assessment modeling in aviation (Lee, 2006)	✓	⨯	✓	✓	⨯	✓
Systematic accident risk assessment by Monte Carlo (Stroeve et al., 2009)	⨯	✓	✓	⨯	✓	⨯
Flight operation risk assessment system (Hadjimichael, 2009)	⨯	✓	✓	⨯	⨯	⨯
Aircraft performance risk assessment model (Cohen et al., 1999)	⨯	⨯	⨯	✓	⨯	⨯
Human factor analysis and classification system (Li et al., 2008)	⨯	⨯	⨯	✓	⨯	⨯
Systems‐theory accident modeling and processes (Allison et al., 2017)	✓	✓	⨯	⨯	✓	✓
ACCIMAP model (Wienen et al., 2017)	✓	✓	⨯	⨯	✓	✓
Functional resonance analysis method (Badhe et al., 2015)	✓	✓	⨯	⨯	✓	✓
**Our proposed model**	✓	✓	✓	✓	✓	✓

Abbreviation: FCM, fuzzy cognitive map.

According to the reviewed previous research and Table 2, there are several research gaps in this scope, which are explained as follows:

1. Lack of a systematic risk assessment model in aviation accident analysis that is compatible and applicable to complex transportation systems, especially commercial aviation, maritime, and railway transport. Introduced models in literature are commonly utilized in a particular domain; therefore, a need for a practical model can be seen.
2. Most of the previous research focused on a confined group of influential factors (e.g., mechanical or human factors) that were effective in the occurrence of accidents.
3. Usually, the interactions between risks are ignored in studies; in addition, the role of each risk and its effects on the other risks are not considered properly.
4. Risk assessment techniques have some shortcomings and limited solutions in dealing with environments with uncertainties. Failure mode and effects analysis (FMEA) and intuitionistic fuzzy‐FMEA (IF‐FMEA) often use multi‐attribute decision making (MADM) techniques to rank risks, and there are no more options.

Due to the vital role of safety in transportation systems and, also, the existence of the mentioned gaps, this research proposes a comprehensive and sequential risk assessment model for the aviation industry, utilizing historical accident data and addressing the complex relationships among factors. It combines risk analysis and soft computing methods, including IF‐FMEA and FCM. Novel prioritization methods are introduced for IF‐FMEA, and they are validated through two MADM techniques, enhancing accuracy in assessing and prioritizing risks. The research emphasizes evaluating system behavior under various scenarios, considering the influence of each risk in the scenario‐creation process.

To address the limitations of traditional FMEA, researchers often combine it with analytical techniques for improved results. This study employs two approaches: intuitionistic fuzzy sets (IFSs) to manage uncertainties and, also, various ranking techniques to enhance the accuracy of outcomes. Employment of IFS and IF‐FMEA instead of other FSs and the FMEA extensions primarily originates from our subject of research and case study. In this way, these two tools have exclusive features and advantages, including the following items that best fit with our research considerations (Behret, 2014; Ghasemi & Rahimi, 2023):

- Complex relationships: IFSs are better suited for modeling complex, multi‐dimensional relationships in data and systems. They can handle scenarios where different variables exhibit different degrees of uncertainty and hesitancy.
- Effective multi‐criteria decision analysis: IFSs are beneficial for multi‐criteria decision analysis, especially when different criteria involve different degrees of uncertainty and hesitation. They offer a more nuanced evaluation of alternatives.
- Scenario analysis: IF‐FMEA is well suited for scenario analysis, enabling the exploration of various “what‐if” scenarios to understand the potential consequences of different risk factors and their interactions.
- Compliance with real‐world ambiguity: In many real‐world situations, ambiguity and uncertainty are inherent. IF‐FMEA's capacity to explicitly handle these factors makes it a practical choice for risk analysis.

Now, it can be said that the main contributions of this study are briefly as follows:

1. A comprehensive and sequential risk assessment model is introduced for analyzing accidents and incidents in crucial transportation systems, especially commercial aviation. This model evaluates all influential risk factors of any type, scores and prioritizes them, represents the interactions and relationships among them, considers uncertainties and ambiguities in the research environment, and can evaluate scenarios and predict the behavior of the system in the various conditions. In addition, FCMs have never been utilized in such a model that gets inputs from other analytical methods like IF‐FMEA in previous studies. It can be said that this model has all the features and requirements needed in this subject.
2. In order to deal with the solution limitations in the IF‐FMEA subject, this research introduces two innovative and novel risk prioritization techniques that work based on the features of both FMEA and IFSs.
3. A particular case study is considered in this article, and the proposed model is implemented completely utilizing real data. The results of this process illustrate the efficiency and application of the constructed model as well as two innovative introduced risk prioritization techniques.

In addition, the employment of FCMs in this study has considerable novelties, including:

1. In the proposed model, FCM gets input from FMEA through a structured and sequential risk assessment model.
2. This study shows how considering the interactions between risks can affect their importance and rank using FCM; moreover, a comparison between the results obtained from FMEA and FCM is presented.
3. The present research uses FCM scenario analysis capability to evaluate the role of each risk, group of risks, and other potential scenarios to analyze the behavior of the system and provide predictive insights.

In the rest of this article, in Section 2, the theoretical foundations of the problem and the utilized techniques are explained. In Section 3, the methodology of the research and the steps in the construction of the mentioned model are introduced; in Section 4, the results of this study will be represented comprehensively; in Sections 5 and 6, conclusions, discussions, and managerial insights are brought.

## 2 UNDERPINNING THEORIES

### 2.1 Intuitionistic fuzzy set theory

IFSs, introduced by Atanassov (1999), extend FSs to handle uncertainties beyond partial belonging, particularly in situations with insufficient information. Unlike traditional FSs, IFSs incorporate both membership and non‐membership to address vagueness more comprehensively. This approach is widely applied across various fields, offering a more nuanced representation of uncertainty.

Definition 2.1.1. An intuitionistic fuzzy sub‐set of reference set X or an IFS in the reference set X is a set like A. For each member, x∈X two values, including membership and non‐membership, are assigned. Therefore, Atanassov's IFS is defined as
(1) $$A=\left\{≺x,\mu_{A}\left(x\right),v_{A}\left(x\right)≻\forall x\in X\right\},$$

where $\mu_{A}\left(x\right),v_{A}\left(x\right)$ are membership and non‐membership values in a way that both are FSs, so IFSs, also known as two‐dimensional FSs. Moreover, these two values are defined as $\mu_{A}\left(x\right):x\to \left[0,\;1\right],\;v_{A}\left(x\right):x\to \left[0,\;1\right]$, and, respectively, $0\leq \mu_{A}\left(x\right)+v_{A}\left(x\right)\leq 1$ are presented. Index $\pi_{A}$ is introduced as a hesitancy value according to the Atanassov IFS and calculated by

(2) $$\pi_{A}=1-\left(\mu_{A}\left(x\right)+v_{A}\left(x\right)\right).$$

Definition 2.1.2. Assume intuitionistic fuzzy numbers $A\;=\{x,\mu_{A}\left(x\right),v_{A}\left(x\right)|x\in X\}$, $A_{1}=\left\{x,\mu_{A1}\left(x\right),v_{A1}\left(x\right)|x\in X\right\}$, $A_{2}=\left\{x,\mu_{A2}\left(x\right),v_{A2}\left(x\right)|x\in X\right\}$, the intuitionistic fuzzy operations are defined as follows:
(3) $$\bar{A}=\left\{≺x,v_{A}\left(x\right),\mu_{A}\left(x\right)≻\forall x\in X\right\},$$
(4) A1∩A2=⟨x,minμA1x,μA2x,μA1maxvA1x,vA1x|x∈X⟩,
(5) A1∪A2=⟨x,maxμA1x,μA2x,μA1minvA1x,vA1x|x∈X⟩,
(6) A1×A2=⟨x,μA1x×μA2x,vA1x+vA2xμA1−vA1x×vA2x|x∈X⟩.

Moreover, for an IFS like Equation (1), the defuzzied value can be obtained using (Atanassova & Sotirov, 2012)

(7) $$\mathrm{Def}=\mu_{A}+\pi_{A}\left(\frac{\mu_{A}}{\mu_{A}+v_{A}}\right).$$

As this study employs the experts’ opinions, there is a need to aggregate them; to this end, the intuitionistic fuzzy weighted aggregation operator presented by Xu (2007) is used and defined as Equation (8). It is assumed that $R^{A}={\left(r_{ij}^{A}\right)}_{m\times n}\;$ is the intuitionistic decision matrix, and $\lambda \;=\{\lambda_{1},\;\lambda_{2},\lambda_{3}\text{…},\;\lambda_{A}\}\;$ are the weight of each decision‐maker so that $\sum_{A=1}^{l}\lambda_{A}=1,\lambda_{A}\in \left[0,1\right]$ is established:

(8) $$\begin{array}{ccc} & & \;r_{ij}=IFWA_{\lambda }\left(r_{ij}^{1},r_{ij}^{2},\text{…},r_{ij}^{l}\right)=\lambda_{1}r_{ij}^{1}⊕\lambda_{2}r_{ij}^{2}⊕\lambda_{3}r_{ij}^{3}\text{…}⊕\lambda_{l}r_{ij}^{l}\\ & & \;=\left[1-\prod_{A=1}^{l}{\left(1-\mu_{ij}^{A}\right)}^{\lambda_{A}},\prod_{A=1}^{l}{\left(v_{ij}^{A}\right)}^{\lambda_{A}},\prod_{A=1}^{l}{\left(1-\mu_{ij}^{A}\right)}^{\lambda_{A}}-\prod_{A=1}^{l}{\left(v_{ij}^{A}\right)}^{\lambda_{A}}\right]. \end{array}$$

Generally, the utilization of IFSs has many considerable advantages in comparison to conventional FSs, especially for the considered case study of this research. The most important advantages of this theory are the following items:

1. In addition to membership values, IFSs determine non‐membership values, which indicates how much an element does not belong to a considered reference set.
2. An IFS determines a value called hesitancy, which indicates that the IFSs are a representation to express the uncertainty in assigning membership degrees to the elements (Lee et al., 2001).

As is said in Section 1, analyzing commercial aviation accidents is seriously complicated, skill‐based, and deals with various uncertainties and vagueness. On the other hand, IFSs are an extension of FSs that can deal with these issues, and they can lead to obtaining more accurate results (Lee et al, 2001). Therefore, this study frequently utilizes this theory to ensure the quality of the outcomes.

### 2.2 Failure mode and effects analysis

FMEA systematically identifies root causes, failure modes, and their relative risks to enhance system reliability, quality, and safety (Xiao et al., 2023). It employs severity (*S*), occurrence (*O*), and detection (*D*) criteria to calculate an RPN, with higher RPN values indicating higher priority risks (Equation 9). Severity assesses the impact of failure, occurrence estimates the probability of a failure mode, and detection gauges the likelihood of identifying a failure, helping to limit or avoid system risks. FMEA also aids in evaluating and optimizing maintenance programs by considering any potential risks within the system:

(9) RPN=S×O×D.

### 2.3 Innovative risk prioritization methods

The study fills the mentioned research gap by introducing new risk prioritization approaches combining IFS theory with FMEA, initially employing traditional MADM methods like TOPSIS and MARCOS, then showcasing the innovative techniques to validate their effectiveness.

#### 2.3.1 Ranking based on intuitionistic fuzzy RPN (IF‐RPN)

First, it should be said that there is a prerequisite to implementing these two methods. That prerequisite is to calculate the final values of the *S*, *O*, and *D* factors for each failure mode (risk). To this end, the process of scoring and aggregating experts’ opinions is introduced in Section 4. Now, in this method, we attempt to utilize the RPN values just like the conventional FMEA.

Therefore, here, due to implementing FMEA in an intuitionistic fuzzy environment, the intuitionistic fuzzy RPN (IF‐RPN) should be calculated. In this way, the mentioned values are computed by multiplying the three mentioned factors using Equation (6). In the conventional FMEA, risks are ranked based on crisp RPN values in descending order, but this does not work for IF‐RPN values. Therefore, now we use the proposed process by Xu (2007) to compare these values and prioritize risks. To this end, the score function *S* is calculated by Equation (10); this value evaluates the degree of suitability that an alternative satisfies a decision‐maker's requirements. Then, the accuracy function *H*, which is the degree of accuracy of intuitionistic fuzzy values, is calculated as well using Equation (11). Finally, failure modes are ranked based on these two functions. If $A=[\mu_{A},1-v_{A}]$ be an intuitionistic fuzzy value, the mentioned functions are calculated as follows:

(10) $$S\left(A_{i}\right)=\mu_{A_{i}}\;-v_{A_{i}},$$

(11) $$H\;\left(A_{i}\right)=\mu_{A_{i}}\;+v_{A_{i}}.$$

#### 2.3.2 Ranking based on the vague sets (RB‐VS)

A vague set is a collection of elements within a universe, each assigned a membership value ranging continuously between 0 and 1. This means that every element in the set can be associated with both true and false membership values. Let us denote this vague set as *V*. Suppose *U* represents the universe of discourse containing *X* objects, where each object has *x* elements. In this context, *V* in *U* can be described using two functions: true membership (*V_t_*) and false membership (*V_f_*). Both *V_t_* and *V_f_* are real numbers within the interval [0, 1]. *V_t_* is the lower bound on the grade of membership of *x* derived in favor of *x*, and *V_f_* is the lower bound on the grade of membership derived against *x* for each element *x* in *X*: $V_{t}+V_{f}\leq 1\;\mathrm{and}\;V_{t}:X\to \left[0,1\right],\;V_{f}:X\to \left[0,1\right]$

Hence, the membership value for *x* is bound to $\left[V_{t}\left(x\right),1-V_{f}\left(x\right)\right]\;\mathrm{of}\;\left[0,1\right]$. To clarify the meaning of the membership value here, let us consider an example in which the severity of a certain risk is obtained as a vague set [0.639, 0.715]; this set indicates a range where the level of severity associated with the risk lies. In a universe of discourse *U*, a vague set *V* is defined by its true membership function, *t_v_*, and its false membership function, *f_v_*, as outlined in the following equations (Badhe et al., 2015):

$$t_{v}:U\to \left[0,1\right],$$

$$f_{v}:U\to \left[0,1\right],\;\mathrm{and}$$

$$t_{v}\left(x\right)+f_{v}\left(x\right)\leq 1,$$

In this method, we implement the proposed process by Chen and Tan (1994) to prioritize risks. In this way, first, the *S*, *O*, and *D* values of each risk should be transformed into its vague set. After that, for each risk, the evaluation function E is calculated using Equation (12) in it; ⋏ and ⋎ are the minimum and maximum operators considering $A_{i}=\left[\mu_{i},t_{i}^{*}\right]$ be a vague set, where $t_{ij}^{*}=\;1-v_{A_{i}}$. Finally, risks are ranked based on the obtained values in descending order:

(12) $$E\left(A_{i}\right)=\left(\left[\mu_{ij},t_{ij}^{*}\right]⋏\left[\mu_{ik},t_{ik}^{*}\right]⋏\text{…}\left[\mu_{ip},t_{ip}^{*}\right]\right)⋎\left[\mu_{is},t_{is}^{*}\right].$$

### 2.4 Fuzzy cognitive maps

**FIGURE 1:**
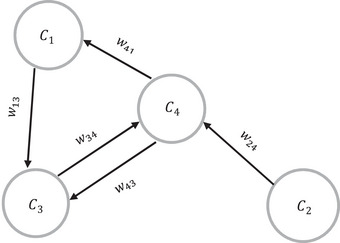
Example of a simple fuzzy cognitive map (FCM).

FCMs, introduced by Kosko (1986), describe relationships between concepts in complex decision making systems, employing nodes and directional causal relationships with weighted arcs (Efe, 2019). Positive weights indicate that an increase in one concept causes an increase in another, whereas negative weights indicate the opposite (Malek, 2017). FCMs provide a framework for analyzing systems and creating unlimited scenarios to evaluate system behavior under various conditions, especially useful in the aviation industry to examine the main root causes of diverse scenarios in aviation accidents. Figure 1 shows a simple example of an FCM with four nodes so that $C_{i}$ show the concepts and $W_{ij}$ represent the relationships among the concepts of considering graph.

The main goal of FCM is to predict the output of the problem by considering the interactions among the concepts. The values of each node are obtained through several iterations until reaching the steady state, using the FCM approach and Equation (13) so that $A_{i}^{k}$ expresses the value of the concept $C_{i}$ in the (*k*)^th^ iteration. $W_{ji}$ is the weight of the directed arc from the node $C_{j}$ to $C_{i}$. f is a threshold function that keeps the values of the nodes in the range [0,1]. Finally, $A_{i}^{k+1}$ is the value of the concept $C_{i}$ in (*k*+1)^th^ iteration:

(13) $$A_{i}^{k+1}=f\left(A_{i}^{k}+\sum_{j=1}^{N}A_{j}^{k}\times W_{ji}\right).$$

## 3 RESEARCH METHODOLOGY

As is said in Section 1, the present article attempts to propose a novel and comprehensive sequential model that is used to analyze accidents in transportation systems, especially in the commercial aviation industry. It should be said that this model is more suitable for accidents related to vehicles with more complex, systematic functions and with a larger number of people involved due to the existence of many different influential factors. It can lead to more accurate and reliable results (especially in commercial aviation, maritime, and railway transport). This sequential model has been established based on various steps that provide inputs for each other, and overall, it is a combination of a risk assessment method (FMEA) and a soft computing technique (FCM), which will work in an intuitionistic fuzzy environment. In this model, accident‐contributing factors are extracted and considered failure modes (risks), and in the next step, they will be the concepts of FCM's system. The mentioned model basically looks to answer two vital questions as follows:

1. How an analytical model can be constructed and operated to evaluate accidents in transportation systems?
2. What changes will happen in the results of the accident analysis procedure when we affect the interaction between concepts of the system?

In the following section, the case study, data collection process, and precise procedure of the proposed accident analysis model will be illustrated.

This study conducts a risk assessment analysis of Iran's commercial aviation accidents, addressing challenges such as financial crises, international sanctions, and an aging fleet. These issues have led to numerous accidents, resulting in 2150 fatalities and substantial financial losses. Despite the insufficient existing research on this topic, this study aims to improve safety through an analytical approach. The Iran Civil Aviation Authority (CAA) manages aviation affairs, whereas the Aviation Accident Investigation Board (AAIB), comprising experts, investigates and reports on accidents under the CAA.

Moreover, the considered case study is implemented to answer the following important questions:

1. What are the most critical risks (e.g., mechanical failures and human errors) that were effective in the occurrence of accidents? To this end, this model uses both intuitionistic fuzzy FMEA utilizing four various prioritizing techniques, and in addition, FCM obtains another ranking considering relationships among the risks.
2. What is the role of each risk in the analyzing system and its influence on the other risks (concepts) due to the existence of relationships and interactions among them? As we know, FCMs provide a valuable framework that will be used by this research to analyze the importance of the concepts in a system; additionally, a lot of scenarios that evaluate the role of each concept are determined as a novel approach.

### 3.1 Data collection

To do this research, the accidents and serious incidents from 1979 to 2021 have been collected precisely and attempted to prevent missing any cases. To this end, the primary resource is the CAA accident database (https://aig.cao.ir/). It should be noted that this database had no final reports for some cases; therefore, other valid resources like the International Civil Aviation Organization (ICAO) database (https://www.icao.int/safety/airnavigation/AIG) and the Aviation Safety Network (ASN) database (https://aviation‐safety.net/database) are utilized to obtain them. The proposed model extracts the required information from historical data; in this case, the official reports of occurrences will be used. In countries with developed commercial aviation networks, some corresponding organizations and institutions are responsible for dealing with unfortunate occurrences. They scrutinize the accidents comprehensively, and at the final phase of analysis, an official report on that occurrence is prepared and sent to the ICAO and society. The mentioned reports are considered the main resource of data in this model that was collected completely.

### 3.2 The proposed risk assessment model

In this research, an expert team comprising eight members, including five specialists from AAIB, an associate professor from Aircraft Maintenance Eng. Dep, Civil Aviation Technology College, Tehran, Iran; a B1 engine and fuselage maintenance technician (Senior) from Iran Air company, and a safety and quality insurance manager, conducted the FMEA process. Additionally, in the FCM process, four experts of AAIB and the mentioned maintenance technician participated.

**FIGURE 2:**
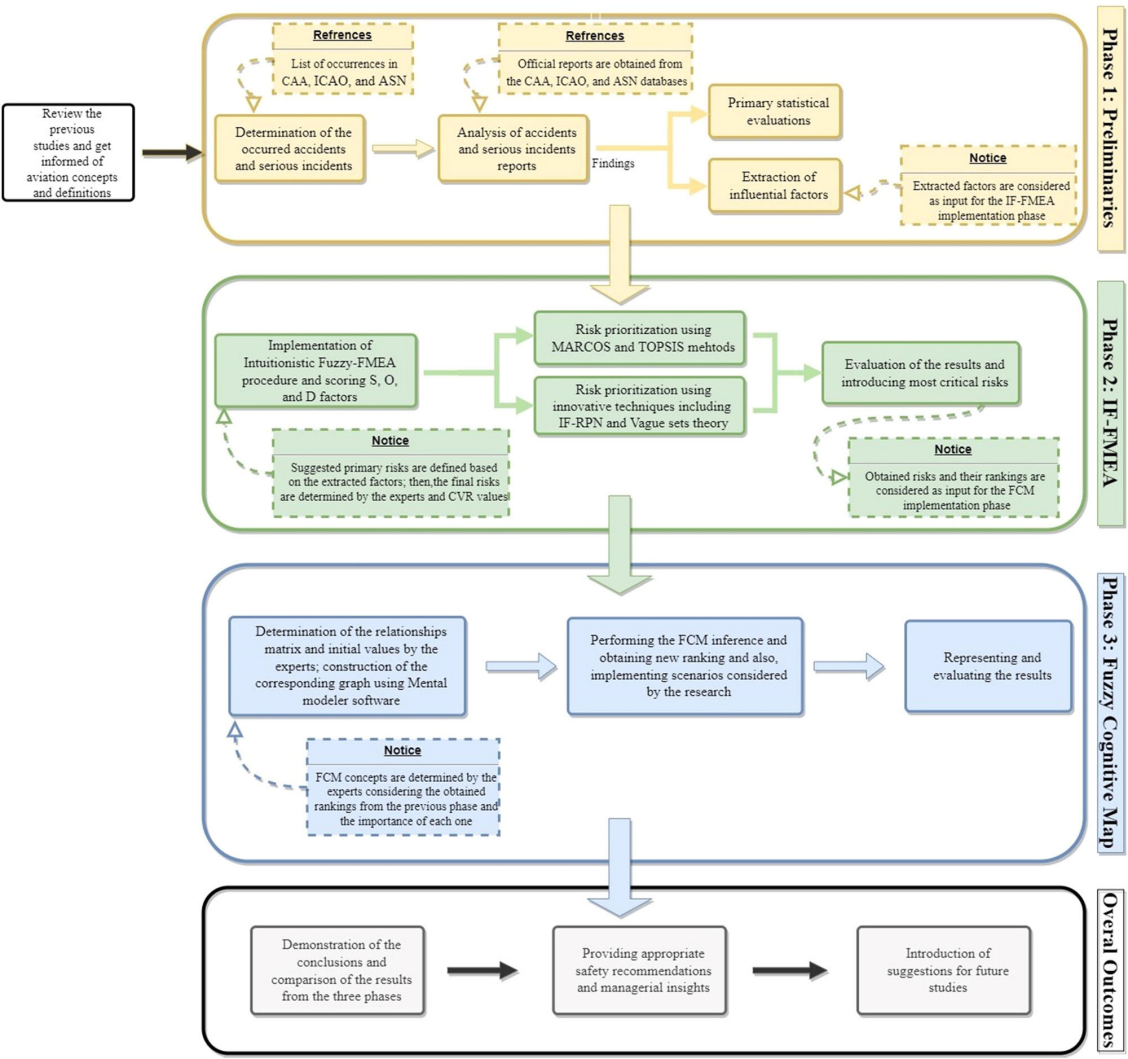
Research procedure.

Overall, the research procedure comprises three major stages. In the first stage, accidents and serious incidents are identified, and official reports are collected and evaluated. In the second stage, the FMEA process is applied based on the first‐stage findings, and in the third stage, an FCM is used to depict relationships within the system and provide different evaluations. The second and third stages are conducted in an intuitionistic fuzzy environment to handle expert uncertainties. Each stage yields outputs that can be used in subsequent stages or considered separate research outcomes. These stages are explained in detail in the following steps; in addition, Figure 2 shows the whole process of research:

***Step* 1**. Due to the complexity and vastness of commercial aviation accidents and incidents, the first step is to review the previous studies and get informed of aviation concepts and definitions.
***Step* 2**. The accidents and serious incidents should be collected precisely. To this end, the existing accidents and serious incident cases in the CAA, ICAO, and ASN databases are compared and gathered according to the date of occurrence. These items form a list that includes the mentioned period's occurrences.
***Step* 3**. To analyze these occurrences, thorough information and details are needed. Therefore, the official reports and data are extracted from the noted resources in the previous step. These reports and data comprehensively explain the accidents and serious incidents from different aspects.
***Step* 4**. The research team thoroughly analyzes reports and data for each accident and serious incident individually, aiming to identify all influential factors contributing to these events, which encompass mechanical failures, human errors, hazardous conditions, and other relevant elements. Experts are consulted to validate and make revisions if necessary.
***Step* 5**. In FMEA sessions with experts, a list of suggested failure modes is provided via a questionnaire, and experts express their opinions on the relevance of each mode to the study. Some failure modes are removed in this step using the content validity ratio (CVR) and Cronbach's alpha values to enhance the accuracy of the analysis. The final selection of failure modes is made after this step.
***Step* 6**. Experts evaluate the severity (S), occurrence (O), and detection (D) factors of failure modes using linguistic variables, and the final values are determined by aggregating their opinions. Four different methods are employed to rank the failure modes, including two MADM techniques (TOPSIS and MARCOS) and two innovative approaches (IF‐RPN and ranking based on vague sets or RB‐VS), providing a comprehensive assessment of the proposed methods’ validity and accuracy.
***Step* 7**. In this step, the most critical failure modes are considered in the concepts of the FCM based on experts’ opinions. Then, they are asked to determine the existence and strength of the relationships among the concepts. The final value of the weight matrix is obtained through the aggregation process.
***Step* 8**. The corresponding graph is drawn and analyzed. Experts determine the initial values of the concepts, the FCM inference is made, and another ranking is obtained again.
***Step* 9**. In this step, the concepts are considered activated separately and one by one in the form of different scenarios; then, the outcomes of each scenario and the effect of each concept on the evaluating system are analyzed.
***Step* 10**. The results of the different stages are evaluated and compared together in this step. The strengths and weaknesses of each method and technique in this scope are shown.
***Step* 11**. In the final step, overall conclusions will be presented. In this way, several appropriate recommendations and vital actions will be stated; moreover, necessary managerial insights and suggestions for future works will be introduced.

## 4 DATA ANALYSIS

The analysis process is done according to the mentioned steps in Section 3.2, and the obtained results are presented in the current section. Additionally, the completed affairs are explained in more detail, and the consequences of this study are presented separately for the three divided stages. This study is structured in such a way that each of the stages somehow is a prerequisite for the next stage, and nothing is based on assumptions. The obtained results are brought in the following sections.

### 4.1 Preliminary findings

**FIGURE 3:**
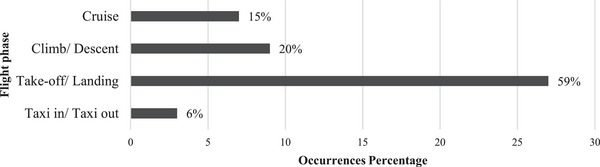
Iran aviation accident and serious incident frequencies based on flight phases.

**FIGURE 4:**
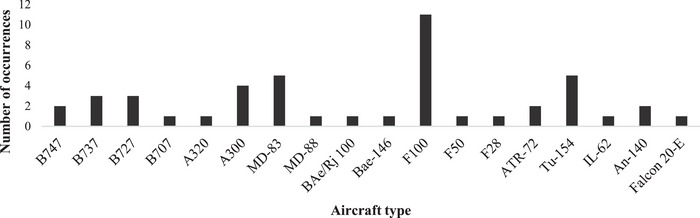
Aircraft types involved in accidents and serious incidents.

The study first identified 46 aviation accidents and serious incidents in Iran from 1979 to 2021, primarily from the CAA database and cross‐referenced with the ICAO and ASN databases. Official final reports for 30 cases were collected from the CAA database, and for the remaining 16 occurrences, only preliminary reports were available, so we also used valid information existing in the ASN and ICAO databases to complete our dataset as required. The focus in this step was on determining influential factors and exploring valuable raw data for insights. The main objective of this effort is to determine the influential factors, as is said in Section 3.2, but there is much valuable raw data that can lead to significant findings. Therefore, the accidents and serious incidents are divided into the flight phases, which means we specify that each occurrence took place in one of the four flight phases. Corresponding frequencies are shown in Figure 3. As expected, the findings showed that take‐offs and landings are the most vulnerable parts of a flight (Honn et al., 2016; O'Connor & Kearney, 2018; Naeeri et al., 2019). The second point is the frequency of each aircraft type in the occurrence of accidents and serious incidents, which are introduced in Figure 4. The findings showed that Fokker 100, Tu‐154, MD83, and A300 are at the top of this ranking.

As said before, the most crucial objective of this section is to determine the influential factors that were effective in the occurrence of accidents and serious incidents. These factors include mechanical failures and malfunctions, human errors and mistakes, weather conditions, and natural phenomena. The research team scrutinized each one of the reports and information to get to the factors during the group sessions. Resources directly mentioned some of these factors, and others were chosen at the researchers’ discretion. Therefore, a list including 86 extracted factors was generated early.

To enhance the accuracy of the results, the supplement required information, and to prevent some factors from being overlooked, three experts with the highest experience (AAIB top manager and deputies) were asked to review the list and eliminate the irrelevant factors, and just in case there were any ignored factors, they could add them to the list. They meticulously did this and added eight new missed factors (19, 28, 34, 53, 73, 76, 81, and 84 in Table A1) and omitted two factors, including “Hostile dialogue between pilot and co‐pilot” and “Lack of secondary check by the maintenance supervisor.” More information about this is explained in Appendix C. Finally, 92 exclusive influential factors were collected, introduced in the mentioned table in Appendix A.

As is seen in Table A1, factors are assigned to four categories, including (a) Human, (b) Mechanical failure and malfunctions, (c) Environmental, and (d) Managerial/Organizational. The “Human” category is related to any human errors and incorrect actions done by the flight crew, air traffic control personnel, maintenance engineers, and so on. The “Mechanical failure and malfunctions” category is associated with any failure or malfunction in any system or sub‐system of the aircraft that leads to an unsafe condition. The “Environmental” category is related to any natural phenomena or artificial facilities that make flights dangerous. Managerial/Organizational category is associated with responsible organizations’ deficiencies, incorrect policies, and so on. Additionally, some of these factors were common in different occurrences; therefore, their repetitions are calculated in the “Frequency” column.

### 4.2 Intuitionistic FMEA and rankings

In this step, 45 failure modes (preliminary list) are defined by the researchers based on the influential factors that were extracted in the previous section (Table A1); then, the experts were asked by a questionnaire to announce their agreement on each of the failure modes as an adequate root cause of occurrences and the necessity of their presence in the investigation process, according to the 9‐point Likert scale (Gharibnejad & Ostadi, 2020). To this end, we calculate the CVR according to the experts’ announcements by Equation (14) (Lawshe, 1975). As said before, our FMEA team had eight members; therefore, the failure modes with CVR less than 0.75 and low Cronbach's alpha values (explained in the next paragraph) were eliminated from the research process:

(14) $$\mathrm{CVR}=\frac{N_{E}-N/2}{N/2}.$$

To put it simply, the failure modes with $N_{E}\geq 7$ will remain in the research process. N is the total number of experts, and $N_{E}$ is the number of experts who distinguished a specific failure mode as “necessary.” According to our Likert scale, scores of 6 and more are considered the necessary values in this study. Finally, 25 failure modes were determined to be used in the FMEA process. As we got to the final failure modes, we distributed questionnaires among the experts through the FMEA sessions. We asked them to rate the failure modes’ *S*, *O*, and *D* factors according to the linguistic variables introduced in Tables B1, B2, B3 (Sayyadi Tooranloo et al., 2018). We modified and customized these scoring scales based on research subjects and experts’ advisements to strengthen the accuracy of judgments. Moreover, in this research to evaluate the reliability of the experts’ judgments and scorings, Cronbach's alpha values are calculated for four categories of failure modes in the preliminary list of 45 failure modes using SPSS software. In addition, an overall value is calculated as well by aggregating all the items from the four categories into one set; the outcomes are shown in Table 3. As is shown in this table, the overall Cronbach's alpha value is more than 0.7, which means the considering reliability is excellent. Here, it should be mentioned that after performing CVR and Cronbach's alpha methods, the factors related to the “Environmental” and “Managerial/Organizational” categories were excluded from the research process because they did not get acceptable values of CVR and Cronbach's alpha, and our model stopped doing further analysis about them.

**TABLE 3:** Cronbach's alpha values.

Category	Human errors	Mechanical failure and malfunctions	Environmental	Managerial/Organizational	Overall value
Cronbach's alpha	0.938	0.921	0.326	0.258	0.962

To clarify the interpretation of scoring scales in the mentioned tables, for example, if an expert determines the severity of a specific risk as “Partly High” with IFS (0.8, 0.1, 0.1), it means that with a degree of 0.8 (which is close to 1), this risk belongs to the set of risks with severe consequences, or strong evidence or judgment is supporting the assessment that the potential consequences of the failure are severe, and also, with a degree of 0.1, this risk does not belong to the mentioned set, or there is minimal evidence or disagreement suggesting that the consequences might not be as severe as initially assessed. In addition, 0.1 interprets a slight hesitation or uncertainty in fully committing to the assessment of severity as a risk with severe consequences.

The experts rated the mentioned factors for each failure mode individually, and the research team supervised this process as facilitators and the FMEA managers. It was tried to establish an appropriate communication space between the experts and handle the vagueness during the sessions. To this end, for all of the scoring scale tables, linguistic terms, and descriptions (e.g., Tables B2 and B3), the research team explained them, clarified ambiguities, answered their questions, and also brought practical examples; in addition to these considerations, all of the meetings were recorded and the audio file was provided to relevant experts so that they could use them in a consistent and reproducible way whenever they needed those explanations. In this study, we calculated the weights of the decision‐makers (*DM_i_*) according to various indicators like their ages, experiences, and expertise, using the intuitionistic fuzzy numbers in Table B1. The final weights of each decision‐maker are introduced in Table 4 using Equation (7). After that, the CVR values and aggregation of experts’ opinions are calculated by Equations (8) and (14). The outcomes are shown in Table B4.

**TABLE 4:** Weights of the decision‐makers.

$DM_{i}$	$DM_{1}$	$DM_{2}$	$DM_{3}$	$DM_{4}$	$DM_{5}$	$DM_{6}$	$DM_{7}$	$DM_{8}$
**Weight**	0.1497	0.1706	0.1706	0.1706	0.1497	0.0999	0.019	0.0699

As an example to clarify scorings, “Failure in the hydraulic system” can be valued by an expert as follows: “High” severity due to its costly damage to aircraft, “Very low” occurrence because it happens on average, about every 1000 flight cycles, and “High” detection because it can be detected easily by indicators and sensors. Now, this study ranks the failure modes using four methods, including two MADM TOPSIS and MARCOS, and two innovative proposed approaches, RB‐VS and IF‐RPN, which are introduced in Sections 2.3.1 and 2.3.2. It should be noted that two innovative proposed methods utilize the intuitionistic fuzzy environment properties to prioritize the options.

To implement MADM procedures, we consider the failure modes as the alternatives and *S*, *O*, and *D* factors as the attributes or criteria. The introduced weights for attributes and the proposed algorithm by Sayyadi Tooranloo et al. (2018) are employed for TOPSIS in this research. It should be noted that we used spherical distance (Abramowitz & Stegun, 1964) to calculate the distance of each alternative from ideal and anti‐ideal values instead of Euclidean distance. In addition, the proposed algorithm by Liu and Li (2021) is used to rank the failure modes based on the intuitionistic fuzzy MARCOS method. In these two MADM methods, the *S*, *O*, and *D* factors are considered positive attributes. According to the referred studies, the failure modes are ranked based on higher values of the proximity ratio ($C_{i}$) and the utility function of alternatives ($f({\overset{ˇ}{K}}_{i})$). The final findings are presented in Table 5.

**TABLE 5:** Outcomes of the failure mode and effects analysis (FMEA) and Spearman test.

	Methods	TOPSIS		MARCOS		IF‐RPN		RB‐VS		Aggregated ranking
	Failure modes	$C_{i}$	Rank	$f({\overset{ˇ}{K}}_{i})$	Rank	$H_{\tilde{a}}$	Rank	$S_{E_{A_{i}}}$	Rank	
Mechanical failures	F_1_	0.5878	5	0.6829	5	0.2244	6	−0.0154	4	5
	F_2_	0.5624	7	0.6734	7	0.2209	7	−0.1073	6	6
	F_3_	0.4608	16	0.6369	10	0.1834	11	−0.0633	5	15
	F_4_	0.5202	12	0.5929	17	0.1233	20	−0.4010	24	19
	F_5_	0.6227	4	0.7206	4	0.2539	4	0.0275	2	4
	F_6_	0.5008	14	0.6447	9	0.1954	8	−0.1894	10	11
	F_7_	0.3446	24	0.5325	23	0.1028	23	−0.3634	21	23
	F_8_	0.5460	10	0.6229	14	0.1570	14	−0.3355	18	14
	F_9_	0.5668	6	0.6342	12	0.1501	16	−0.2845	17	9
	F_10_	0.3720	21	0.5528	22	0.1045	22	−0.3786	22	22
	F_11_	0.5520	9	0.6366	11	0.1738	13	−0.1837	9	10
Human errors	F_12_	0.8964	1	0.8442	1	0.4252	1	0.1097	1	1
	F_13_	0.5132	13	0.6129	15	0.1855	9	−0.2170	12	12
	F_14_	0.7346	3	0.7938	2	0.3603	2	0.0229	3	2
	F_15_	0.7432	2	0.7395	3	0.2800	3	−0.2038	11	3
	F_16_	0.4488	18	0.5865	19	0.1282	18	−0.3852	23	16
	F_17_	0.4835	15	0.6261	13	0.1748	12	−0.2598	14	13
	F_18_	0.4460	19	0.5994	16	0.1524	15	−0.2827	15	17
	F_19_	0.2806	25	0.5161	24	0.0978	24	−0.3487	19	24
	F_20_	0.4523	17	0.5837	20	0.1267	19	−0.2252	13	18
	F_21_	0.3617	22	0.5530	21	0.1148	21	−0.3525	20	21
	F_22_	0.4297	20	0.5871	18	0.1344	17	−0.2842	16	20
	F_23_	0.5591	8	0.6824	6	0.2301	5	−0.1371	7	7
	F_24_	0.5349	11	0.6503	8	0.1835	10	−0.1602	8	8
	F_25_	0.3503	23	0.5026	25	0.0496	25	−0.6759	25	25
**Methods**	**TOPSIS**	1		0.925		0.872		0.734		**Spearman test results**
	**MARCOS**	0.925		1		0.970		0.884		
	**IF‐RPN**	0.872		0.970		1		0.894		
	**RB‐VS**	0.734		0.884		0.894		1		
***N* = 25 and Correlation is significant at 0.01 level (2‐tailed)**		**Correlation coefficient**								

Abbreviations: IF‐RPN, intuitionistic fuzzy risk priority number; RB‐VS, ranking based on the vague sets.

We considered an innovative approach for the following ranking method that first calculates the IF‐RPN according to Equations (6) and (9). After that, based on the proposed method by Xu (2007), the score function ($S_{\tilde{a}}$), which evaluates the degree of suitability that an alternative satisfies a decision‐maker's requirements, and accuracy function ($H_{\tilde{a}}$), which evaluates the degree of accuracy of the intuitionistic fuzzy value $\tilde{a}$ are calculated, and the failure modes are ranked. For the following method, we considered the features of the vague sets (Gau & Buehrer, 1993) for prioritizing the failure modes according to the proposed approach by Xu (2007). In this method, values of the score function ($S_{E_{A_{i}}}$) are used to evaluate and rank the options. The final findings of these two methods are shown in Table 5 as well. Moreover, in the last column, we determined the final rank of each failure mode using the Copeland ranking aggregation method (Lestari et al., 2018).

According to Table 5, it can be said that the obtained ranks for each failure mode from various methods are almost close together, especially for the top five critical failure modes. Nevertheless, some differences are seen, which can be justified due to the different features and approaches of the utilized methods. The Spearman's rank correlation test conducted using SPSS software revealed a significant correlation among the rankings presented in Table 5 as well. The results indicated that the two groups of ranking methods performed nearly identically in terms of performance and accuracy. Additionally, the novel proposed methods, which employ intuitionistic fuzzy characteristics, were utilized to demonstrate their functionality and benefits in the case study. These methods also provided additional solutions for IF‐FMEA utilization. Furthermore, the comparison results obtained from the MADM in the innovative proposed methods affirmed their validity and accuracy. Based on the mentioned table, F_12_, F_14_, F_15_, F_5_, and F_1_ are the most critical failure modes effective in Iran's commercial aviation accidents and serious incidents. The first three failure modes are related to human errors and mistakes; the others are mechanical failures and malfunctions. As we know, the torsion link component, breaks, and anti‐skid system belong to the landing gear system, but due to their critical role in studied accidents and serious incidents, these items are considered separate failure modes in FMEA to obtain deeper evaluations. Finally, five failure modes are dedicated to the aircraft's landing system (F_1_, F_3_, F_5_, F_6_, and F_11_). It should be noted that F_1_ in this research refers to failures of any other component in the landing gear system except the mentioned items. More evaluations of the critical failure modes and several safety actions will be stated in Sections 5 and 6.

### 4.3 Fuzzy cognitive map utilization

In commercial aviation, accidents result from complex interactions of numerous factors, each affecting the others. Accidents are often the culmination of a series of sequential, minor factors such as failures and errors. Understanding these relationships and counteractions among influential factors is crucial in enhancing aviation safety during accident investigations. As said in Section 2.4, FCMs are valuable tools for modeling complex systems, such as aircraft crashes. This study combines FMEA and FCM methods to improve accuracy and comprehensiveness. After the FMEA process, consulting with experts helped to identify 15 critical failure modes, enhancing the system's behavior representation by reducing the number of risk factors. In the next step, the experts built their individual FCMs, which means they evaluated the relationships among the concepts and examined the initial values of the concepts according to their importance based on linguistic variables in Table 6 (Mirghafoori et al., 2018). If all experts announced the “No Influence” for the relationship among two considered concepts, the corresponding value is assigned 0 in the relationship's matrix.

**TABLE 6:** Intuitionistic fuzzy linguistic terms.

Linguistic terms	IFNs
Negligible importance/Influence	(0.1, 0.9, 0)
Low importance/Influence	(0.35, 0.6, 0.05)
Medium importance/Influence	(0.5, 0.45, 0.05)
High importance/Influence	(0.75, 0.2, 0.05)
Very high importance/Influence	(0.9, 0.1, 0)

Moreover, as said in the previous section, five experts participated in the FCM procedure, who are again weighted in Table 7. The outcomes for initial values and relationship matrix are obtained using Equations (7) and (8) and shown in Tables 8 and 9. The selected concepts for FCM belong to mechanical failures and human errors.

**TABLE 7:** The weights of the experts.

$DM_{i}$	$DM_{1}$	$DM_{2}$	$DM_{3}$	$DM_{4}$	$DM_{5}$
**Weight**	0.231	0.231	0.202	0.134	0.202

**TABLE 8:** The concepts and initial values.

	Failure mode	Concept	Description	Initial values	
				Fuzzy intuitionistic	Crisp
Mechanical failures	F_1_	$C_{1}$	Failure in the landing gear system	(0.702,0.259,0.039)	0.730
	F_2_	$C_{2}$	Failure in the engines and losing power	(0.752,0.225,0.023)	0.769
	F_3_	$C_{3}$	Failure in the hydraulic system	(0.622,0.325,0.053)	0.657
	F_5_	$C_{5}$	Failure and fracture in torsion link component	(0.513,0.433,0.054)	0.542
	F_6_	$C_{6}$	Failure in the anti‐skid system	(0.464,0.481,0.055)	0.491
	F_8_	$C_{8}$	Fracturing the first‐row disk of the engine's low‐pressure compressor	(0.306,0.656,0.038)	0.318
	F_9_	$C_{9}$	Failure and loss in flight control surfaces	(0.815,0.166,0.019)	0.831
Human errors	F_11_	$C_{11}$	Failure in break system	(0.358,0.598,0.044)	0.374
	F_12_	$C_{12}$	Pilot error: landing with inappropriate speed and altitude	(0.623,0.324,0.053)	0.658
	F_13_	$C_{13}$	Pilot error: excessive pitch‐up while landing	(0.405,0.545,0.050)	0.426
	F_14_	$C_{14}$	Pilot error: not following the instructions and taking inappropriate actions	(0.681,0.286,0.033)	0.704
	F_15_	$C_{15}$	Pilot error: attempting to land at an unauthorized airport	(0.276,0.687,0.037)	0.286
	F_17_	$C_{17}$	Pilot error: incorrect decision making	(0.579,0.366,0.054)	0.613
	F_23_	$C_{23}$	Pilot error: fly despite fatigue and high workload	(0.754,0.213,0.032)	0.779
	F_24_	$C_{24}$	Technical personnel error: wrong set up of hydraulic pipes in the anti‐skid system	(0.218,0.755,0.026)	0.224

**TABLE 9:** Relationships matrix.

	$C_{1}$	$C_{2}$	$C_{3}$	$C_{5}$	$C_{6}$	$C_{8}$	$C_{9}$	$C_{11}$	$C_{12}$	$C_{13}$	$C_{14}$	$C_{15}$	$C_{17}$	$C_{23}$	$C_{24}$
$C_{1}$		0.287	0.539	0.822	0.775	0	0	0.730	0.349	0.301	0.488	0.306	0.403	0.314	0
$C_{2}$	0.386		0.345	0.402	0	0.656	0.345	0.268	0.468	0.473	0	0.226	0.374	0.268	0
$C_{3}$	0.468	0		0.452	0.431	0.278	0.659	0.345	0.557	0.511	0.338	0	0.319	0.268	0
$C_{5}$	0.855	0	0.643		0.676	0	0.224	0.632	0.338	0.295	0	0	0.402	0	0
$C_{6}$	0.402	0	0	0.409		0	0	0.586	0.300	0.234	0.226	0	0.280	0	0.339
$C_{8}$	0.338	0.884	0.392	0	0		0.350	0	0.569	0.436	0.319	0.299	0.452	0.168	0
$C_{9}$	0.386	0	0.386	0.287	0	0		0	0.654	0.734	0.404	0	0.452	0.268	0
$C_{11}$	0.386	0	0.484	0.320	0.569	0	0		0.386	0.338	0.278	0	0.392	0.268	0
$C_{12}$	0.771	0.319	0.319	0.545	0.417	0	0	0.436		0.649	0.426	0.286	0.549	0.224	0
$C_{13}$	0.705	0	0.553	0.463	0.368	0	0	0.392	0.318		0	0	0.312	0	0
$C_{14}$	0.513	0.333	0.333	0.374	0.253	0.224	0.294	0	0.738	0.629		0.676	0.658	0.653	0
$C_{15}$	0.386	0	0	0.253	0	0	0	0	0.488	0	0.512		0.572	0.403	0
$C_{17}$	0.299	0	0	0.294	0	0	0	0.312	0.676	0.468	0.683	0.511		0.482	0
$C_{23}$	0.426	0	0	0.294	0	0	0	0	0.565	0.410	0.586	0.404	0.771		0
$C_{24}$	0.261	0	0.555	0.261	0.839	0	0	0.320	0.234	0	0.261	0	0	0	

Now the initial values and relationship matrix are obtained. In the next step, the FCM evaluations will be done based on the experts’ opinions. To this end, we used FCM Expert software (Nápoles et al., 2018) to draw the corresponding graph and implement the FCM inference. Moreover, various scenarios are defined and analyzed. For the preliminary analysis of the obtained FCM, several indices, such as indegree, outdegree, and centrality, are commonly defined (Tchupo, 2018). These indices present valuable information about each risk factor and the system's overall condition, as shown in Table 10. According to this table, the corresponding density value is more than 0.5 and refers to a high‐density network. Moreover, it indicates many connections among the concepts; therefore, the failure modes and influential factors are severely and widely related. The connection per component value also proves this fact.

**TABLE 10:** Fuzzy cognitive map (FCM) indices.

	Total components: 15	Total connections: 134	Density: 0.6381	Connection per component: 8.933
	Failure modes	Indegree	Outdegree	Centrality
Mechanical failures	$C_{1}$	6.5820	5.314	11.896
	$C_{2}$	1.823	4.211	6.034
	$C_{3}$	4.549	4.626	9.175
	$C_{5}$	5.176	4.065	9.241
	$C_{6}$	4.328	2.776	7.104
	$C_{8}$	1.158	4.207	5.365
	$C_{9}$	1.872	3.571	5.443
	$C_{11}$	4.021	3.421	7.442
Human errors	$C_{12}$	6.634	4.941	11.581
	$C_{13}$	5.478	3.111	8.589
	$C_{14}$	4.521	5.678	10.199
	$C_{15}$	2.708	2.614	5.322
	$C_{17}$	5.936	3.725	9.661
	$C_{23}$	3.316	3.456	6.772
	$C_{24}$	0.339	2.731	3.07

In Table 10, the biggest values of indegrees belong to *C*_1_ and *C*_12,_ which means that the other concepts strongly influence these two concepts. Any changes in the concepts can lead to considerable variation in the two mentioned concepts. On the other hand, *C*_1_ and *C*_14_ have the biggest values of outdegrees, which means these concepts strongly affect the others. Any changes in the two mentioned concepts can lead to massive variations in the other concepts of the system. Overall, *C*_1_, *C*_12_, and *C*_14_ are the central concepts of our FCM graph with the highest values of centrality; sometimes, this index is used to introduce the most important concepts of a system, but in this study, we use other precise tools to this end. The centrality index merely presents the most effective and impressible factors in an overall view.

**FIGURE 5:**
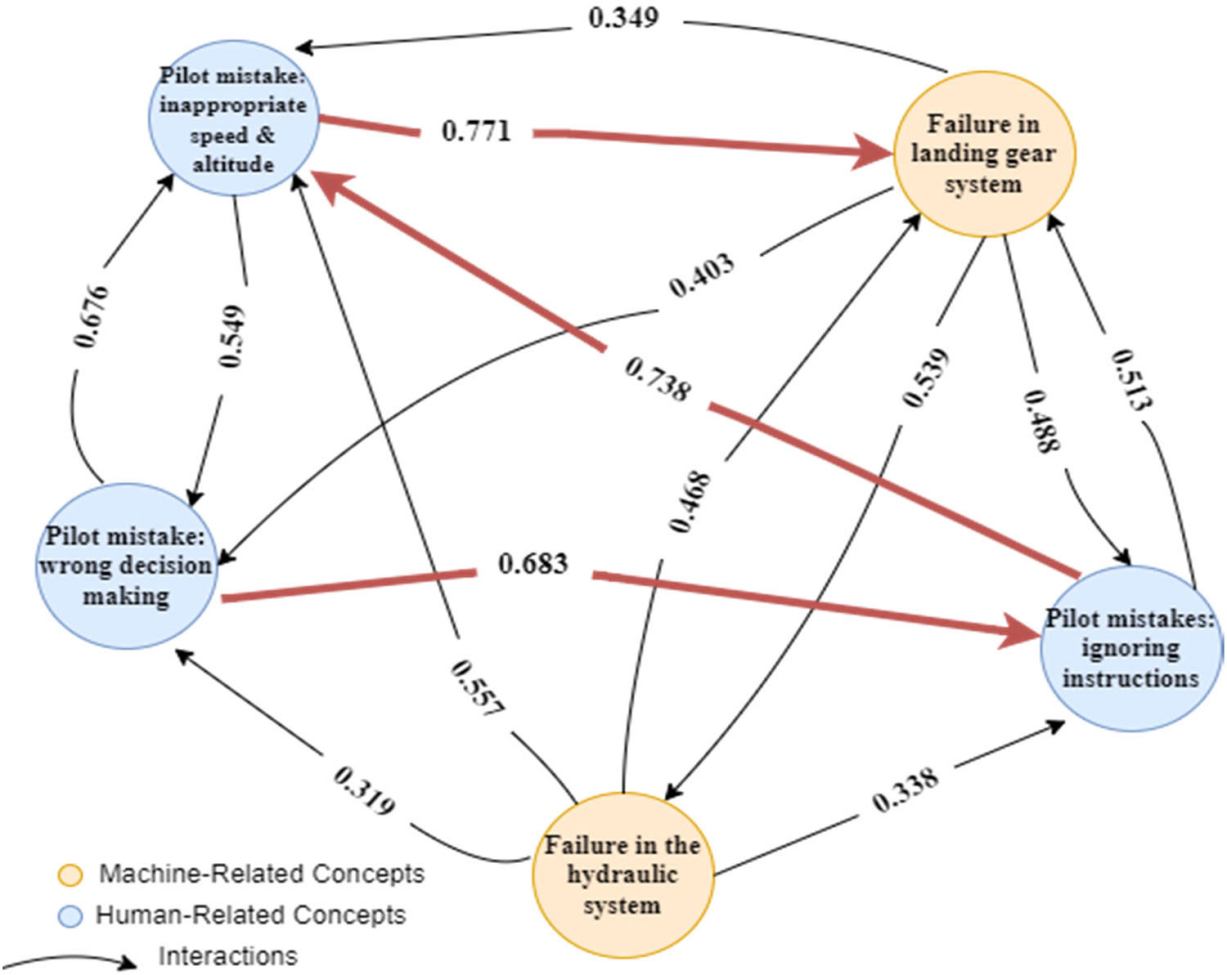
Concepts with the highest impacts.

Now, to get detailed in the obtained values of indices in Table 10, Figure 5 shows the concepts with the highest impact in our system (highest values of indegree, outdegree, and centrality) and the considerable interactions among them. As is seen, the most important interactions are highlighted with red; thus, it can be perceived that inappropriate speed and altitude of the plane almost always happen by the pilot, especially during landing (*C*_12_), which can lead to severe damage to the aircraft landing gear system. In addition, one of the most important causes of this mistake is the ignoring of instructions again by the pilot, according to Figure 5. Additionally, these outcomes conclude that wrong decision making is one of the most effective concepts in evaluating our system.

In this step, risk factors (concepts) are prioritized, considering the relationships among them and using FCM inference. It should be noted that the FCM Expert software is used to calculate the final values of each concept in the procedure of the FCM inference; moreover, we configured it to do it based on the FCM inference classic method (Equation 13). In this procedure, the sigmoid function is considered the transfer or threshold function, and the iteration stop condition to reach a fixed‐point attractor is considered 0.001. The outcomes are presented in Table 11. According to this table, Failure in the landing gear system (*C*_1_), Pilot error: landing with inappropriate speed and altitude (*C*_12_), and Pilot error: incorrect decision making (*C*_17_) are the most critical risk factors of Iran's commercial aviation accidents and incidents. The inference process and FCM indices introduce almost the same critical factors, especially for the first and second ranks.

**TABLE 11:** The fuzzy cognitive map (FCM) inference.

Step	Mechanical failures								Human errors						
	*C*_1_	*C*_2_	*C*_3_	*C*_5_	*C*_6_	*C*_8_	*C*_9_	*C*_11_	*C*_12_	*C*_13_	*C*_14_	*C*_15_	*C*_17_	*C*_23_	*C*_24_
0	0.730	0.769	0.657	0.542	0.491	0.318	0.831	0.374	0.658	0.426	0.704	0.286	0.613	0.779	0.224
1	0.988	0.846	0.957	0.975	0.938	0.762	0.878	0.936	0.989	0.982	0.964	0.888	0.984	0.942	0.596
2	0.999	0.920	0.993	0.997	0.992	0.858	0.929	0.991	0.999	0.998	0.994	0.969	0.999	0.983	0.714
3	0.999	0.932	0.995	0.998	0.994	0.877	0.938	0.993	0.999	0.998	0.995	0.974	0.999	0.985	0.741
4	0.999	0.934	0.995	0.998	0.994	0.880	0.939	0.993	0.999	0.998	0.995	0.974	0.999	0.986	0.746
5	0.999	0.934	0.995	0.998	0.994	0.880	0.939	0.993	0.999	0.998	0.995	0.974	0.999	0.986	0.747
6	0.999	0.934	0.995	0.998	0.994	0.880	0.939	0.993	0.999	0.998	0.995	0.974	0.999	0.986	0.747
**Rank**	1,2	13	7	5	8	14	12	9	1,2	4	6	11	3	10	15

As is said already, aviation accidents often have unique sequences of events but share common key factors like mechanical failures or human errors. In this research, the focus is on evaluating crucial scenarios that can occur at any time. Using the FCM's ability to define and assess various scenarios (Alipour et al., 2019), the study examines the impact of each concept, representing key factors, on the problem, considering their presence and activation across different scenarios, which are explained in the following. The utilization of the FCM Expert software in this research was done exactly based on the classic FCM features explained in Section 2.4.

**FIGURE 6:**
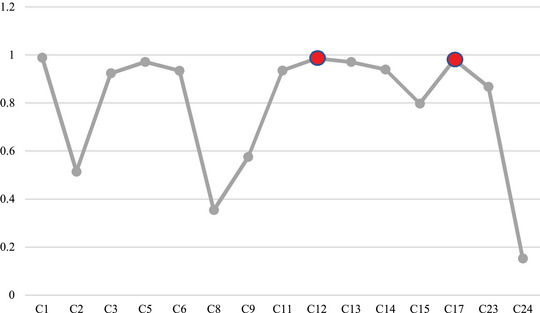
Result of Scenario 1.

**FIGURE 7:**
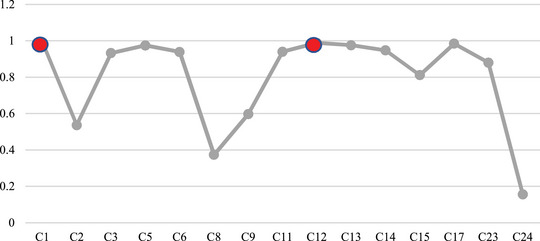
Results of Scenario 10.

**Group Scenarios 1–15, activating each risk factor individually—**In this group of scenarios, we attempt to analyze the role of each risk factor (concept) in our considered system and its effects on the other risks. Moreover, by this means, it is possible to show how a single mechanical failure or human error can exacerbate others and finally lead to an unfortunate occurrence through sequential events. The initial values in these vectors can be interpreted as the severity or activation level of the corresponding concept. In this way, we activate a single concept in each scenario and perform FCM inference using FCM expert software, considering the hyperbolic as a transfer function and slope 0.5, and epsilon 0.1. For example, in the first scenario, $C_{1}$ will be activated; therefore, the initial value vector will be like A=[0.73000000000000000]. We will implement this procedure for each concept in further scenarios one by one. Figures 6 and 7 are examples that show the behavior of this group of scenarios and the concepts with the most changes.

**Scenario 16 (** $G_{A}$)—In this scenario, the risk factors related to mechanical failures and malfunctions are considered active, and concepts related to human errors and mistakes are considered inactive. The initial values of the concepts in this scenario are considered A=[0.7300.7690.6570.5420.4910.3180.8310.3740000000].

**Scenario 17 (** $G_{B}$) —In this scenario, the risk factors related to human errors and mistakes are considered active, and concepts related to mechanical failures and malfunctions are deemed inactive. The initial values of the concepts in this scenario are considered A=[000000000.6580.4260.7040.2860.6130.7790.224].

**Scenario 18 (** $G_{C}$) —Here, due to the important role of the landing gear system in our system, the risk factors related to the landing gear system (*C*_1_, *C*_3_, *C*_4_, *C*_5_, *C*_8_) are considered active, and others are inactive. The initial value vector of the concepts in this scenario is considered A=[0.73000.6570.5420.491000.3740000000].

It should be noted that the initial value vectors of each scenario are presented in Table 12, and the final results are introduced in Table 13. Moreover, more comprehensive evaluations and analyses are presented in Section 5. The obtained results from the mentioned 18 scenarios in Table 13 point out that the activation of certain risk factors, like “Fracturing the first‐row disk of the engine's low‐pressure compressor (*C*_8_),” “Failure and loss in flight control surfaces (*C*_9_)”, and “Pilot error: fly despite fatigue and high workload (*C*_23_),” can significantly impact other factors, potentially leading to accidents. Conversely, risk factors “Pilot error: attempting to land at an unauthorized airport (*C*_15_)” and “Technical personnel error: wrong setup of hydraulic pipes in the anti‐skid system (*C*_24_)” have the least influence on the system. Mechanical failures are shown to be linked to staff errors, such as incorrect decision making and inappropriate landing speeds (*C*_12_ and *C*_17_). External factors can affect pilots’ decisions, making it crucial to eliminate factors that impact decision making. Furthermore, mechanical failures can complicate landings as this flight phase involves the engagement of multiple aircraft systems and components, making defect prevention essential.

**TABLE 12:** The initial values in each scenario.

Scenario	Mechanical failures								Human errors						
	*C*_1_	*C*_2_	*C*_3_	*C*_5_	*C*_6_	*C*_8_	*C*_9_	*C*_11_	*C*_12_	*C*_13_	*C*_14_	*C*_15_	*C*_17_	*C*_23_	*C*_24_
1	0.730	0	0	0	0	0	0	0	0	0	0	0	0	0	0
2	0	0.769	0	0	0	0	0	0	0	0	0	0	0	0	0
3	0	0	0.657	0	0	0	0	0	0	0	0	0	0	0	0
4	0	0	0	0.542	0	0	0	0	0	0	0	0	0	0	0
5	0	0	0	0	0.491	0	0	0	0	0	0	0	0	0	0
6	0	0	0	0	0	0.318	0	0	0	0	0	0	0	0	0
7	0	0	0	0	0	0	0.831	0	0	0	0	0	0	0	0
8	0	0	0	0	0	0	0	0.374	0	0	0	0	0	0	0
9	0	0	0	0	0	0	0	0	0.658	0	0	0	0	0	0
10	0	0	0	0	0	0	0	0	0	0.426	0	0	0	0	0
11	0	0	0	0	0	0	0	0	0	0	0.704	0	0	0	0
12	0	0	0	0	0	0	0	0	0	0	0	0.286	0	0	0
13	0	0	0	0	0	0	0	0	0	0	0	0	0.613	0	0
14	0	0	0	0	0	0	0	0	0	0	0	0	0	0.779	0
15	0	0	0	0	0	0	0	0	0	0	0	0	0	0	0.224
16	0.730	0.769	0.657	0.542	0.491	0.318	0.831	0.374	0	0	0	0	0	0	0
17	0	0	0	0	0	0	0	0	0.658	0.426	0.704	0.286	0.613	0.779	0.224
18	0.730	0	0.657	0.542	0.491	0	0	0.374	0	0	0	0	0	0	0

**TABLE 13:** Results of scenarios.

		Mechanical failures (risk factors)								Human errors (risk factors)						
Scenario	Description	$C_{1}$	$C_{2}$	$C_{3}$	$C_{5}$	$C_{6}$	$C_{8}$	$C_{9}$	$C_{11}$	$C_{12}$	$C_{13}$	$C_{14}$	$C_{15}$	$C_{17}$	$C_{23}$	$C_{24}$
1	Activating $C_{1}$	0.9891	0.5137	0.9238	0.971	0.9342	0.3535	0.5748	0.9353	0.9854	0.9706	0.9394	0.7974	0.9808	0.8672	0.1519
2	Activating $C_{2}$	0.9882	0.5056	0.92	0.969	0.9316	0.3436	0.5653	0.9326	0.984	0.9683	0.9358	0.791	0.9791	0.8613	0.1498
3	Activating $C_{3}$	0.9913	0.5393	0.9333	0.9751	0.9397	0.3767	0.6	0.9407	0.9887	0.976	0.948	0.8134	0.9847	0.8813	0.1563
4	Activating $C_{5}$	0.9903	0.5271	0.9289	0.9733	0.9373	0.3647	0.5879	0.9382	0.9873	0.9736	0.9441	0.806	0.9829	0.8748	0.1544
5	Activating $C_{6}$	0.991	0.5358	0.9321	0.9746	0.939	0.3729	0.5964	0.94	0.9883	0.9753	0.947	0.8113	0.9842	0.8795	0.1558
6	Activating $C_{8}$	0.9911	0.5369	0.9325	0.9748	0.9392	0.3742	0.5976	0.9402	0.9884	0.9756	0.9473	0.812	0.9843	0.8801	0.1559
7	Activating $C_{9}$	0.9913	0.5404	0.9337	0.9753	0.9399	0.3776	0.6011	0.9409	0.9888	0.9762	0.9484	0.814	0.9848	0.8819	0.1564
8	Activating $C_{11}$	0.9909	0.534	0.9315	0.9744	0.9387	0.3714	0.5948	0.9397	0.9881	0.975	0.9464	0.8103	0.9839	0.8786	0.1555
9	Activating $C_{12}$	0.988	0.5044	0.9193	0.9688	0.9312	0.3429	0.5638	0.9323	0.9837	0.9679	0.9351	0.7899	0.9787	0.8604	0.1496
10	Activating $C_{13}$	0.9911	0.5364	0.9323	0.9747	0.9391	0.3736	0.5971	0.9401	0.9884	0.9755	0.9472	0.8117	0.9843	0.8798	0.1559
11	Activating $C_{14}$	0.9895	0.5177	0.9252	0.9716	0.935	0.3564	0.5784	0.9361	0.986	0.9714	0.9407	0.7998	0.9814	0.8693	0.1563
12	Activating $C_{15}$	0.0853	0.0312	0.0497	0.0756	0.0573	0.0082	0.0148	0.0594	0.0863	0.0899	0.0729	0.0754	0.0835	0.0599	0
13	Activating $C_{17}$	0.9905	0.529	0.9296	0.9736	0.9377	0.3668	0.5898	0.9387	0.9875	0.974	0.9448	0.8072	0.9832	0.8759	0.1547
14	Activating $C_{23}$	0.9911	0.5368	0.9324	0.9748	0.9392	0.3742	0.5976	0.9402	0.9884	0.9755	0.9473	0.8117	0.9843	0.8801	0.1559
15	Activating $C_{24}$	0.0702	0.0132	0.035	0.0635	0.0539	0.0119	0.028	0.0637	0.059	0.0592	0.0388	0.0181	0.0585	0.0302	0.0159
16	Activating $G_{A}$	0.9909	0.5385	0.9319	0.9743	0.9386	0.3703	0.596	0.9396	0.9882	0.9752	0.9467	0.8106	0.984	0.8787	0.1553
17	Activating $G_{B}$	0.9893	0.5174	0.9243	0.9712	0.9342	0.3547	0.5765	0.9353	0.9857	0.971	0.9402	0.7994	0.9812	0.8686	0.1516
18	Activating $G_{C}$	0.9911	0.537	0.9326	0.9748	0.9394	0.3743	0.5978	0.9403	0.9885	0.9756	0.8474	0.8121	0.9844	0.8802	0.1561

In scenario $G_{B}$, we showed that human errors, especially pilots, can lead to intense damage to the components of aircraft engaged in the landing phase. It is probably because the landing procedure is done by the pilots manually most of the time when they do not use the instrument landing system. This fact may increase the chance of error in their performance, so usually these errors are considered the root causes of the mechanical failure appearance. It should be noted that these human errors, in most cases, cause damage through the chain of events and mistakes. Moreover, in this study, a logical relationship can be seen between the results of the scenarios $G_{A}$ and $G_{B}$. During scenario execution, the values of concepts in an FCM change significantly at each step of the inference process. Once a steady state is reached, these final values represent the study's outcomes, primarily indicating an increase in risk factor values. These changes can be seen as the generation, amplification, or reinforcement of affected concepts within the FCM.

## 5 DISCUSSION

A comprehensive discussion of the analysis is presented as follows:

1. According to Section 4.1, with a statistical view, most of the accidents and serious incidents occurred during the take‐offs and landings; in addition, considering the obtained rankings from the FMEA and FCM techniques in Sections 4.2 and 4.3, the most critical failure modes that were introduced by the utilized techniques are directly related to take‐off and landing phases of a flight, as expected. Therefore, “landing with inappropriate speed and altitude” is presented by the research as the most critical failure mode in the corresponding system. Generally, this failure mode is related to human errors and mistakes that are made by pilots. Specifically, it occurs when an aircraft tries to land at an airport with an air speed more or less than the determined landing speed; or when an airplane tries to land at an airport with an inappropriate descent rate, for example, a descent gradient of more or less than 3°. This risk factor can cause hazardous occurrences like tail strikes and runway excursions. It can also lead to severe damage to aircraft components, like the landing gear system and fuselage. The main reasons for this failure mode are bad weather conditions like heavy winds, the weakness of pilots’ skills, mechanical problems, and, especially, it can be said that failure mode “Pilot error: not following the instructions and taking inappropriate actions” (F_14_) is one of the most important causes of this issue, which is explained in the next paragraph.
2. According to Table 5, “Pilot error: not following the instructions and taking inappropriate actions” (F_14_), mentioned in the previous paragraph, is one of the critical factors in the safety of flights; additionally, it is ranked second in the FMEA ranking. Generally, commercial aviation is established based on the instructions and safety requirements. It means that there are extensive instructions and requirements for any operation that should be considered by the staff precisely. Any inattention to these items can endanger the flight. Some significant causes for this failure mode are a lack of appropriate staff training, individual vices like excessive pride, overconfidence, fatigue, emergency conditions like severe mechanical failures, and a lack of proper cockpit resource management. According to Table 6, “Failure in the landing gear system” (*C*_1_) is ranked second in the FCM ranking. The landing gear system in modern aircraft consists of a main landing gear system in the middle of the fuselage and a nose landing gear system in the forepart of the fuselage. As is declared in Section 4.2, landing gear in this risk factor refers to any component in landing gear systems except the torsion link component, breaks, and anti‐skid system, which are considered single‐failure modes. The primary causes of this failure mode are improper repair or maintenance, failure or fatigue of the components, and failures in other related systems like hydraulic and electric. Moreover, F_14_ can be one of the leading causes of this failure mode.
3. The rankings obtained in the FMEA process, as shown in Table 5, demonstrate relative values for most failure modes. Although there are some minor discrepancies, particularly in the ranking based on vague sets, the differences are infrequent and can be attributed to the nature of the ranking methods used. Overall, there are no significant variations in the rankings presented in Table 5, and the minor differences observed are mathematically justifiable and negligible.
4. This study introduced two innovative methods for prioritizing failure modes in FMEA within an intuitionistic fuzzy environment. The validity of these methods was demonstrated by comparing them with conventional MADM techniques, and they offer several advantages. These innovative methods align directly with the traditional FMEA procedure, providing a structured and straightforward solution, and they use interval values for factors like *S*, *O*, and *D*. Using interval estimates in risk assessment reduces the vulnerability to errors that may arise from depending on single, potentially inaccurate point estimates. As a result, this approach enhances the robustness of risk prioritization (Huang & Xiao, 2021). Notably, this research marks the first application of IF‐RPNs and RB‐VS for prioritizing risks in intuitionistic fuzzy FMEA.
5. The FCM analysis in Section 4.3 revealed that all causal relationships within the system are positive, as expected when dealing with failure modes. The FCM convergence was achieved in six iterations. Results indicate that “Failure in the landing gear system” (*C*_1_) and “Pilot error: not following the instructions and taking inappropriate actions” (*C*_12_) are jointly ranked as the most critical concepts, with the highest centrality index values. These two risk factors are the most crucial contributors to accidents and serious incidents in Iran's commercial aviation system, and their occurrence significantly impacts other risk factors, potentially leading to the emergence and exacerbation of various risks.
6. In scenarios 1–15, we activated each concept one by one and evaluated their role and effects on the considering system and other concepts. As was shown, the risk factor “Pilot error: attempting to land at an unauthorized airport” ($C_{15})$ and “Technical personnel error: wrong set up of hydraulic pipes in the anti‐skid system” $\left(C_{24}\right)$ have the lowest and “Fracturing the first‐row disk of the engine's low‐pressure compressor” $\left(C_{8}\right)$, “Failure and loss in flight control surfaces” $\left(C_{9}\right)$, and “Pilot error: fly despite fatigue and high workload”$\;(C_{23})$ have the most influence on the other concepts of the system. Moreover, we detected that the risk factors “Failure in the landing gear system” ($C_{1})$, “Pilot error: not following the instructions and taking inappropriate actions” $\left(C_{12}\right)$, and “Pilot error: incorrect decision making” ($C_{17})$ are strongly affected by other factors.
7. At the end of Section 4.3, three more scenarios were defined to evaluate the different behaviors of our system. As we know, the failure modes and, afterward, the concepts were assigned to two major categories: human errors and mechanical failures and malfunctions. Therefore, this study dedicated scenarios 16 and 17 to analyze these two categories separately. To this end, the scenario $G_{A}$ considers the machine‐related concepts active; this scenario made a massive change in the values of “Pilot error: not following the instructions and taking inappropriate actions” (*C*_12_), “Pilot error: incorrect decision making” (*C*_17_), and “Pilot error: excessive pitch‐up while landing” (*C*_13_). This finding means that mechanical failures can directly cause several defects in the operation of the aircraft. In Iran's aviation case, the mentioned operation can be stated as a landing process. In addition, making appropriate decisions is a vital duty of the pilots; any incorrect decision can lead to a hazard for flight; therefore, pilots are expected to be ready to make proper decisions in good or bad conditions. Here, it is seen that mechanical failures significantly increase the chance of making wrong decisions (*C*_17_). The scenario $G_{B}$ considers the human‐related concepts active. This scenario caused a massive change in the values of “Failure in the landing gear system” (*C*_1_), “Failure and fracture in torsion link component” (*C*_5_), and “Failure in the hydraulic system” (*C*_3_). It means that the errors and mistakes of the aviation staff, especially the pilots, can severely damage the aircraft landing gear systems in Iran's civil aviation industry. Finally, due to the critical role of the landing gear system in the safety of Iran's air transportation network, scenario 18 considers the activation of the concepts related to failures of the landing gear system. This scenario showed a considerable change in the values of *C*_12_, *C*_17_, and *C*_13_. This scenario obtained the same results as scenario 1; therefore, these failures require special attention.

Iran's aviation system faces challenges, particularly managerial deficiencies. Here, prioritizing safety over financial gains and suggesting a need for a transformative shift in organizational culture and managerial vision in Iran's aviation industry should be emphasized. Given the industry's sensitivities, strict adherence to established rules and instructions is crucial at all organizational levels. The management sector in Iran's aviation system plays a pivotal role in ensuring the enforcement and supervision of these regulations.

## 6 CONCLUSIONS

This study innovatively extracts failure modes directly from official reports, differentiating from previous research relying solely on expert knowledge. It addresses the complex interactions in the aviation industry by employing intuitionistic FCMs, aiming to enhance the accuracy of outcomes in risk assessment. The comprehensive model proposed for evaluating commercial aviation accidents, utilizing accident reports, is adaptable to other transportation systems. The model serves various purposes, including prioritizing risk factors, evaluating relationships, and analyzing countless scenarios to understand system behavior in diverse events. Furthermore, the research introduces two novel risk prioritization methods within the intuitionistic fuzzy FMEA technique, integrating conventional FMEA procedures with features of intuitionistic fuzzy systems theory. To expand on this work, the study recommends future research focus on the technical evaluation of critical failure modes identified and the analysis of root causes for human errors, employing psychological techniques.

## APPENDIX A

### A.1

**TABLE A1:** Influential factor description.

Category	No.	Factor	Frequency
**Human**	1	Pilot error: landing with inappropriate speed and altitude	4
	2	Pilot and flight dispatcher error: carrying excessive fuel	1
	3	Pilot error: wrong decision in selecting landing runway despite tailwinds and incorrect approach	1
	4	Cockpit resource management deficiencies	17
	5	Pilots error: using dual input control	1
	6	Pilots error: excessive pitch‐up during landing	1
	7	Defense system operator error: recognizing the aircraft as a hostile missile	1
	8	Technicians’ weaknesses and mistakes	5
	9	Inappropriate maintenances	2
	10	Pilot error: not following the instructions and doing inappropriate action	15
	11	The pilot was not familiar with the destination airport	1
	12	Pilot error: not following ATC instructions	3
	13	ATC error: wrong scheduling of arrival and departure flights	3
	14	Pilot error: incorrect decision making	4
	15	ATC error: not changing the landing runway in a required situation	1
	16	Pilot error: unauthorized descending	1
	17	Pilot error: making stall condition and not doing required actions	2
	18	Pilot error: using autopilot after a stall condition occurred	1
	19	Maintenance personnel errors: incorrect actions and lack of experience	2
	20	Ground personnel error: lack of runway inspection as requirements and notices	2
	21	Pilot fatigue due to non‐standard flight scheduling	2
	22	Lack of supervision of the technical unit and disregard for the manufacturer's publications	3
	23	Pilot error: returning the lever of the emergency landing wheels to the stowed position	1
	24	Pilot error: adjusting the power of the engines without initial stabilization	1
	25	Pilot error: late take‐off rejection and stopping of the airplane	1
	26	Pilot error: inappropriate usage of engine thrust reverse	3
	27	Pilot error: not notifying the engine pressure ratio (EPR) amount	1
	28	Overconfidence of pilot	1
	29	Pilot error: incorrect calculation of the flight total weight	1
	30	Pilot error: rolling below the speed limit	1
	31	Pilot error: deviating from taxi lines	1
	32	Pilot error: making unreliable airspeed condition	1
	33	Pilot error: not reacting on time against engine failure	1
	34	Putting pressure on the airplane due to excessive take‐off and landing, and maneuvering during training flights	1
	35	Pilot error: attempting to land despite losing part of the runway	3
	36	Pilot error: not locking the thrust reverse system	1
	37	Pilot error: weakness in navigating and proceeding through the approach plan	3
	38	Pilot error: not extending gears while landing	1
	39	Pilot error: deactivating of primary alert system	1
	40	Individual vices of pilots	2
**Mechanical failure and malfunctions**	41	Main landing gear failure	6
	42	Engine high pressure turbine (HPT) failure	1
	43	Hydraulic system failure	3
	44	Very high frequency omnidirectional range station (VOR) system failure	1
	45	Entry of foreign debris into landing gear hydraulic system during maintenance	1
	46	Engine failure and losing power	4
	47	Landing gear shimmy damper component failure	1
	48	Torsion link fracture and failure	1
	49	Nose landing gear fracture	4
	50	Cracks on the nose landing gear due to the fatigue	1
	51	Selector valve leakage and its combination with hydraulic fluid	1
	52	Gears anti‐skid system failure	2
	53	Expansion of cracks due to the vibration caused by the imbalance of the engine HPT module	1
	54	Engine electric control failure	1
	55	Failure of the manual engine start system	1
	56	Landing gears manual extension failure	2
	57	Aircraft altimeter system failure	1
	58	Inappropriate operation of the engine trim	1
	59	Pilot heating system failure	1
	60	Fracture of the first‐row disk of the low‐pressure engine compressor due to fatigue cracking	1
	61	Losing the flight control system	1
	62	Rupture of engine fuel pipe	1
	63	Tire explosion	2
	64	Inappropriate operation of breaks system	1
	65	Detachment of horizontal rudder	1
**Environmental**	66	Downwind	1
	67	Bad weather conditions and low visibility	7
	68	Big windspeed changes	2
	69	Mountain‐wave phenomena	1
	70	Existence of icing clouds	1
	71	Non‐standard conditions of Mehrabad International Airport	1
	72	Too much air traffic in Mehrabad airport	1
	73	Non‐standard parking area	1
	74	Existence of a hook barrier as an obstacle and the lack of lights and warning signs	1
	75	Lack of appropriate navigation systems	1
	76	Mountainous areas near the airport	1
	77	Mehrabad radar and navigation systems failure	1
**Managerial/Organizational**	78	Weakness of protocols and risk management of civilian flights in war alert conditions and lack of proper management	1
	79	Aircraft design deficiencies	2
	80	Lack of appropriate staff training about wind‐shear	1
	81	Lack of area position navigation (APN) system	1
	82	Problems in supplying necessary spare parts and information due to international sanctions	2
	83	The weakness of the airline in training and explaining the instructions and approaches to the pilot, especially about stabilizing the engines before take‐off. Airline weaknesses in monitoring and supervising maintenance	1
	84	Inappropriate and confusing aircraft flight manual (AFM) of the aircraft	1
	85	Lack of appropriate supervision in the calibrating process	1
	86	Aircraft old instruments	1
	87	Lack of proper flight test	1
	88	Manufacturer inappropriate instructions about engine thrust reverse	1
	89	Failure to send the necessary instructions on time about engine inspection by the manufacturer	1
	90	Organization fault in planning the training flights	1
	91	Airline fault in not decommissioning a specific aircraft type	1
	92	The organization's manager's error in ignoring the manufacturer's notices	1

Abbreviation: ATCs, air traffic controls.

## APPENDIX B

### B.1

**TABLE B1:** The rating scale for severity (*S*).

Effect	Statement	Intuitionistic fuzzy number
Extremely high	Severe consequences, high fatality, complete destruction of the plane, without warning	(1, 0, 0)
Very high	Severe consequences, high fatality, complete destruction of the plane, with warning	(0.9, 0.1, 0)
Partly high	Irreparable consequences, severe human damages like maiming, substantial damage to aircraft, and making it useless	(0.8, 0.1, 0.1)
High	Intense consequence, intense human damage, severe and costly damage to aircraft	(0.7, 0.2, 0.1)
Moderate	Considerable consequence, significant human damage requires special care, and great damage to aircraft needs substantial repair	(0.6, 0.3, 0.1)
Low	Average consequence, medium human damages that require outpatient actions, finite damage to aircraft	(0.5, 0.4, 0.1)
Partly low	Light consequence, light human damage like swellings and bruises, light and superficial damage to aircraft	(0.4, 0.5, 0.1)
Very low	Slight consequences, intangible human damage like anxiety, negligible damage to aircraft	(0.25, 0.6, 0.15)
Extremely low	Negligible consequence, lack or very slight human damage, inconsiderable damage to aircraft	(0.1, 0.75, 0.15)
None	No damage to humans and aircraft	(0.1, 0.9, 0)

**TABLE B2:** The rating scale for occurrence (*O*).

Likelihood	Statement	Intuitionistic Fuzzy Number
Imminent	On average, more than 100 cases in each of the 1000 flight cycles	(1, 0, 0)
Very high	On average, about 50 cases in each of the 1000 flight cycles	(0.9, 0.1, 0)
Partly high	On average, about 20 cases in each 1000 flight cycles	(0.8, 0.1, 0.1)
High	On average, about 5 cases in each 1000 flight cycles	(0.7, 0.2, 0.1)
Average	On average, about 3 cases in each 1000 flight cycles	(0.6, 0.3, 0.1)
Low	On average, about 2 cases in each 1000 flight cycles	(0.5, 0.4, 0.1)
Very low	On average, 1 case in each 1000 flight cycles	(0.4, 0.5, 0.1)
Unlikely	On average, 0.5 cases in each 1000 flight cycles	(0.25, 0.6, 0.15)
Very unlikely	On average, 0.1 cases in each 1000 flight cycles	(0.1, 0.75, 0.15)
Impossible	On average, 0.01 cases in each 1000 flight cycles	(0.1, 0.9, 0)

**TABLE B3:** The rating scale for detection (*D*).

Detectability	Statement	Intuitionistic fuzzy number
Impossible	Absolutely undetectable	(0.9, 0.1, 0)
Very low	Insignificant chance of detection	(0.75, 0.2, 0.05)
Average	Fifty–fifty chance of detection	(0.5, 0.45, 0.05)
High	High chance of detection	(0.35, 0.6, 0.05)
Imminent	Absolutely detectable	(0.1, 0.9, 0)

**TABLE B4:** Intuitionistic failure mode and effects analysis (FMEA).

	No.	Failure mode	CVR	S	O	D
Mechanical failures	F_1_	Failure in the landing gear system	1	(0.7249, 0.2012, 0.0739)	(0.5164, 0.3607,0.1229)	(0.3549, 0.6000, 0.0451)
	F_2_	Failure in the engines and losing power	1	(0.6931, 0.1968, 0.1100)	(0.4702, 0.4223, 0.1075)	(0.3942, 0.5619, 0.0440)
	F_3_	Failure in the hydraulic system	0.75	(0.5178, 0.3619, 0.1203)	(0.4890, 0.3895, 0.1214)	(0.4000, 0.5536, 0.0464)
	F_4_	Stall condition	0.75	(0.9328, 0.0570, 0.0102)	(0.2855, 0.5830, 0.1314)	(0.2598, 0.7054, 0.0347)
	F_5_	Failure and fracture in torsion link component	0.75	(0.7047, 0.2247, 0.0705)	(0.3716, 0.5045, 0.1240)	(0.5740, 0.3986, 0.0273)
	F_6_	Failure in the anti‐skid system	0.75	(0.6502, 0.2684, 0.0814)	(0.4101, 0.4590, 0.1308)	(0.3725, 0.5114, 0.0460)
	F_7_	Failure in engine electric control	0.75	(0.6624, 0.2657, 0.0719)	(0.3293, 0.5822, 0.0885)	(0.2731, 0.6909, 0.0360)
	F_8_	Fracturing the first‐row disk of the engine's low‐pressure compressor	0.75	(0.8713, 0.1049, 0.0237)	(0.3290, 0.5494, 0.1215)	(0.3182, 0.6415, 0.0403)
	F_9_	Failure and loss in flight control surfaces	0.75	(0.9286, 0.0640, 0.0075)	(0.2205, 0.6388, 0.1407)	(0.3875, 0.5694, 0.0432)
	F_10_	Rupture of engine fuel pipe	0.75	(0.7629, 0.1622, 0.0748)	(0.2061, 0.6704, 0.1235)	(0.3349, 0.6244, 0.0407)
	F_11_	Failure in breaks system	0.75	(0.8088, 0.1559, 0.0353)	(0.4182, 0.4602, 0.1209	(0.3064, 0.6544, 0.0392)
Human errors	F_12_	Pilot error: landing with inappropriate speed and altitude	1	(0.9040, 0.0800, 0.0161)	(0.6140, 0.3108, 0.0751)	(0.5087, 0.4351, 0.0561)
	F_13_	Pilot error: excessive pitch‐up while landing	0.75	(0.6932, 0.2374, 0.0694)	(0.3884, 0.4893, 0.1223)	(0.3438, 0.6107, 0.0455)
	F_14_	Pilot error: not following the instructions and taking inappropriate actions	1	(0.7591, 0.1622, 0.0787)	(0.5441, 0.3433, 0.1125)	(0.5301, 0.4136, 0.0563)
	F_15_	Pilot error: attempting to land at an unauthorized airport	1	(0.9123, 0.0786, 0.0091)	(0.4405, 0.4393, 0.1202)	(0.4298, 0.5193, 0.0509)
	F_16_	ATC error: wrong planning for arrival and departure flights	0.75	(0.8271, 0.1110, 0.0620)	(0.2496, 0.6082, 0.1421)	(0.3215, 0.6395, 0.0391)
	F_17_	Pilot error: incorrect decision making	0.75	(0.6566, 0.2673, 0.0761)	(0.3794, 0.5024, 0.1182)	(0.4000, 0.5536, 0.0464)
	F_18_	Pilot error: bounce and tail strike occurrences during landing	0.75	(0.6676, 0.2590, 0.0734)	(0.3578, 0.5278, 0.1144)	(0.3602, 0.5922, 0.0475)
	F_19_	ATC error: no changing the corresponding landing runway	0.75	(0.5452, 0.3505, 0.1044)	(0.3207, 0.5567, 0.1226)	(0.2930, 0.6690, 0.0381)
	F_20_	Pilot error: runway incursion occurrence	0.75	(0.7770, 0.1607, 0.0623)	(0.3909, 0.4818, 0.1273)	(0.2420, 0.7315, 0.0265)
	F_21_	Ground personnel error: not measuring the breaking action and informing the crew	0.75	(0.6593, 0.2715, 0.0692)	(0.2614, 0.6083, 0.1303)	(0.3474, 0.6070, 0.0456)
	F_22_	Pilot error: incorrect calculation of total flight weight	0.75	(0.7251, 0.1944, 0.0805)	(0.2697, 0.6377, 0.0925)	(0.3872, 0.5687, 0.0441)
	F_23_	Pilot error: fly despite fatigue and high workload	0.75	(0.6695, 0.2181, 0.1123)	(0.4229, 0.4543, 0.1227)	(0.4556, 0.4932, 0.0511)
	F_24_	Technical personnel error: wrong set up of hydraulic pipes in the anti‐skid system	0.75	(0.7663, 0.1739, 0.0598)	(0.2928, 0.5851, 0.1220)	(0.4555, 0.4952, 0.0492)
	F_25_	Pilot error: activating thrust reverse during flight	0.75	(0.9547, 0.0381, 0.0072)	(0.1031, 0.8032, 0.0937)	(0.2379, 0.7316, 0.0305)

Abbreviation: CVR, content validity ratio.

## APPENDIX C

### Complementary information

In the fourth step of the methodology of this research (introduced in Section 3.2), after collecting official reports of designated accidents, the research team began to study and examine each of them one by one. In this step, the initial list consisting of 86 influencing factors was identified, which, according to them, played a small or large role in the occurrence of the accidents. However, to ensure that all the factors are identified, nothing has been missed, and no irrelevant and unimportant factors have been extracted during this process, the initial list was given to three experts who had the highest experience and expertise. After review, consultation, and consensus, they announced that it is necessary to add eight new factors (19, 28, 34, 53, 73, 76, 81, and 84 in Table A1) to this list and remove two factors from it. Therefore, the final list of factors influencing the occurrence of accidents in our case study consisted of 92 factors. These factors are introduced below and in Table A1 in Appendix A.

**Added factors**:

– Maintenance personnel errors: incorrect actions and lack of experience
– Overconfidence of pilot
– Putting pressure on the airplane due to excessive take‐off and landing, and maneuvering during training flights
– Expansion of cracks due to the vibration caused by the imbalance of the engine HPT module
– Non‐standard parking area
– Mountainous areas near the airport
– Lack of APN system
– Inappropriate and confusing AFM of the aircraft

**Removed factors**:

– Hostile dialog between pilot and co‐pilot
– Lack of secondary check by the maintenance supervisor
